# Difficult-to-diagnose diabetes in a patient treated with cyclophosphamide – the contradictory roles of immunosuppressant agents: a case report

**DOI:** 10.1186/s13256-018-1925-3

**Published:** 2018-12-10

**Authors:** Manuel García-Sáenz, Daniel Uribe-Cortés, Claudia Ramírez-Rentería, Aldo Ferreira-Hermosillo

**Affiliations:** 1 Servicio de Endocrinología, Hospital de Especialidades, Centro Médico Nacional Siglo XXI, Instituto Mexicano del Seguro Social, Cuauhtémoc 330, Colonia Doctores, 06720 Mexico City, Mexico; 2 Unidad de Investigación Médica en Endocrinología Experimental, Hospital de Especialidades, Centro Médico Nacional Siglo XXI, Instituto Mexicano del Seguro Social, Cuauhtémoc 330, Colonia Doctores, 06720 Mexico City, Mexico

**Keywords:** Cyclophosphamide, Diabetic ketoacidosis, Lupus erythematosus, systemic

## Abstract

**Background:**

Cyclophosphamide may induce autoimmune diabetes through a decrease in suppressor T cells and increase of proinflammatory T helper type 1 response in animal models. In humans, this association is not as clear due to the presence of other risk factors for hyperglycemia, but it could be a precipitant for acute complications.

**Case presentation:**

A 31-year-old Mestizo-Mexican woman with a history of systemic lupus erythematosus presented with severe diabetic ketoacidosis, shortly after initiating a multi-drug immunosuppressive therapy. She did not meet the diagnostic criteria for type 1 or type 2 diabetes and had no family history of hyperglycemic states. She persisted with hyperglycemia and high insulin requirements until the discontinuation of cyclophosphamide. After this episode, she recovered her endogenous insulin production and the antidiabetic agents were successfully withdrawn. After 1 year of follow up she is still normoglycemic.

**Conclusion:**

Cyclophosphamide may be an additional risk factor for acute hyperglycemic crisis. Glucose monitoring could be recommended during and after this treatment.

## Background

Most patients with diabetes mellitus (DM) are classified into the commonly accepted groups: type 1 DM (T1DM), type 2 DM, gestational DM, latent autoimmune diabetes of the adult (LADA), or maturity-onset diabetes of the young (MODY). However, almost 10% of patients may prove difficult to classify, especially in the younger groups [1].

T1DM is usually associated with other autoimmune diseases. In this type of diabetes the CD4+ and CD8+ T cells play an important role in the initiation and progression of the disease. The recruitment and activation of these lymphocytes stimulates further secretion of inflammatory cytokines that accelerate beta cell destruction, resulting in insulin depletion [2]. Due to the lack of insulin and the effect of counterregulatory hormones, diabetic ketoacidosis (DKA) may occur [3].

Cyclophosphamide (CY) is a cytotoxic chemotherapeutic agent used in the treatment of hematological diseases. A possible relation between CY and T1DM has been suggested in experimental animal models due to its immunomodulatory properties. It has been shown to promote susceptibility to T1DM in young prediabetic non-obese diabetic (NOD) mice [2, 4–7]. Although the mechanism of action is not clear, some reports have suggested that CY may cause the destabilization of the local immune-regulatory balance by temporarily depleting suppressor cells. Paradoxically, CY has been used in addition to other immune modulators as a treatment for type B insulin resistance syndrome [8, 9].

We present the case of a patient with systemic lupus erythematosus (SLE), not previously diagnosed as having diabetes and with no evidence of autoimmune DM, who developed DKA after six cycles of CY therapy.

## Case presentation

A 31-year-old Mestizo-Mexican woman presented to our emergency department (ED) with DKA. She did not have a family history of diabetes or previous autoimmune diseases. She mentioned multiple hospitalizations due chronic idiopathic pancreatitis between 12 and 16 years of age. At that time, laboratory tests ruled out the presence of gallstones, tumors, autoimmune diseases, or metabolic diseases. Her pancreatic exocrine and endocrine functions were completely normal after each episode. Fifteen years after the last episode of pancreatitis, she presented with alopecia, malar rash, and oral ulcers. She was initially diagnosed as having discoid lupus with the skin biopsy results. However, a week later, she developed severe neuropsychiatric manifestations of SLE, including focal motor seizures, with a Systemic Lupus Erythematosus Disease Activity Index (SLEDAI) score of 18 points. Rheumatologists prescribed methylprednisolone (three doses of 1 g daily by intravenous injection) with improvement of neurological symptoms. Her treatment at hospital discharge was: chloroquine (225 mg/day), levetiracetam (1.5 g twice a day), phenytoin (100 mg twice a day), lorazepam (1 mg/day), and a monthly bolus of CY. In addition, she was treated with weekly down-titrating doses of prednisone (initial dose of 1 mg/kg).

Seven days after administration of the sixth dose of CY (cumulative dose of 6.25 g), she presented with asthenia, adynamia, polydipsia, nausea and vomiting, food intolerance, impaired visual acuity, and abdominal pain. The physical examination at our ED revealed tachycardia (125 beats per minute), tachypnea (27 breaths per minute), drowsiness, and dehydration. She presented with a normal body mass index and no stigmata associated with insulin resistance. Laboratory tests reported: serum glucose of 1240 mg/dl, sodium of 127 mEq/L, potassium of 5.56 mEq/L, chlorine of 86 mEq/L, arterial pH of 7.07, bicarbonate of 3.00 mmol/L, and ketonuria (80 mg/dl) with effective serum osmolarity of 322.8 mOsm/kg and total osmolarity of 334.1 mOsm/kg, with an anion gap of 43.56, urea 63 mg/dl, and creatinine of 1.58 mg/dl. Liver tests, amylase, and lipase were normal. After the diagnosis of a mixed hyperglycemic state with predominance of severe DKA, she was started on an aggressive intravenously administered fluid schedule with saline solution 0.9% and intravenous continuous insulin infusion (CII). During the first day she required a total of 165 units of insulin per day (2.75 U/kg per day). Table 1 shows the clinical and biochemical evolution from first evaluation to resolution of DKA.

**Table 1:** Clinical and biochemical evolution of the patient from arrival to discharge

	Initial evaluation	6 hours*	10 hours*	16 hours*
GCS	12	14	15	15
Vital signs, HR/RR	125/27	117/24	109/19	92/18
Serum glucose (mg/dl)	1240	408	313	174
Urinary ketones (mg/dl)	80	80	40	0
Serum sodium (mEq/L)	127	150	149	152
Serum potassium (mEq/L)	5.56	4.27	3.52	3.5
pH	7.07	6.99	7.21	7.27
Bicarbonate (mmol/L)	3.0	9.2	12.4	14.7
Anion gap	43.56	33.07	25.12	7.04
Osm (mOsm/kg)	322.8	322.6	315.3	313.6
IIR (UI/kg per hour)	0.18	0.1	0.09	0.05

Where: * since initial evaluation, *GCS* Glasgow Coma Scale, *HR* heart rate (in beats per minute), *IIR* insulin infusion rate, *Osm* osmolarity, *RR* respiratory rate (in breaths per minute)

During the first hours, traditional precipitant factors for DKA, such as infections or cardiac ischemia were ruled out. With an aliquot from the first serum sample in the ED, we measured anti-insulin, anti-GAD65, and anti-IA2 antibodies that were reported as negative; C-peptide was determined to be 0.5 ng/ml and the glycated hemoglobin (HbA1c) was 12.0%. The diagnostic consideration was of T1DM, so once DKA remission was achieved, she was prescribed an intensive insulin scheme at a dose of 1.09 IU/kg per day with 70% of basal insulin glargine daily and 30% of lispro boluses divided in three doses.

With the multi-drug immunosuppressive treatment her symptoms improved and 3 months after discharge the CY was switched to azathioprine and then to chloroquine. Three weeks after the switch from CY to other immunosuppressive therapies, she presented with symptomatic hypoglycemia and insulin doses were tapered down. Five months after the switch, insulin was completely withdrawn. At last follow up, 1 year after the hyperglycemic crisis, she had no symptoms related to hyperglycemia and her blood glucose remains under 100 mg/dl. She continues to have a well-balanced, low-carbohydrate diet and her last HbA1c was 5.2% and C-peptide was 1.8 ng/ml without any antidiabetic drugs.

## Discussion

Atypical diabetes accounts for up to 10% of cases of DM. A differential diagnosis is important in order to provide the best initial and long-term management, especially in patients with systemic comorbidities in which glucose elevations may jeopardize their quality of life and overall wellbeing. Initially, in this case, we had four diagnostic possibilities: drug-induced DM (due to prednisone that affects the metabolism of carbohydrates); ketosis-prone diabetes; type 1b DM, and finally a possible association with CY.

The use of glucocorticoids is widely associated with hyperglycemia. They stimulate the activity of phosphoenolpyruvate carboxykinase (PEPCK) in the liver and decrease its activity in the adipose tissue. This enzyme participates in liver gluconeogenesis and increases the level of glycerol 3-phosphate, increasing triacylglycerol (TAG) levels. In the adipose tissue, the inhibition of PEPCK decreases glyceroneogenesis, also decreasing TAG formation. Both, the increase in liver TAG and decrease in adipose tissue TAG formation induce serum elevation of free fatty acids, which are involved in the decrease of sensitivity to insulin in peripheral tissues with a consecutive rise in blood sugar [10]. However, at the time of evaluation, our patient was decreasing her prednisone dose. It should be noted that previously, a dose of high-dose intravenously administered steroids did not cause hyperglycemia. Furthermore, a glucocorticoid rarely precipitates DKA and patients with chronic treatment with a glucocorticoid also develop dermatological manifestations of insulin resistance such as acanthosis nigricans or acrochordons [11], which were absent in this case.

Another entertained diagnosis was ketosis-prone diabetes. This type of diabetes was described in the 1960s in patients who maintain glycemic control without insulin therapy after an episode of DKA. These patients were obese and had a family history of diabetes, preserved insulin secretion, low prevalence of beta cell autoimmunity, and the ability to discontinue insulin therapy [12]. At the time of this publication (18 months after KDA), our patient seems to have recovered her β cell function completely, her weight is normal, she lacks clinical manifestations of insulin resistance, and she does not have a family history of diabetes, thus lacking the classical phenotype for this type of diabetes. In addition, the long-term recovery of endogenous insulin secretion (demonstrated with normal C-peptide levels) and the absence of pancreatic islet autoantibodies leads us to rule out T1DM.

After ruling out all common causes of diabetes, we considered that the hyperglycemic crisis may have been related to the CY treatment or precipitated by it. Previous reports of insulin-dependent DM in non-obese mice [13] also noted that CY, a cytostatic drug used for neoplastic and inflammatory diseases, may have a contradictory effect on immune regulation, affecting even the pancreas and insulin. At high doses it has been used for immunosuppression and at low doses it has been associated with enhanced immune responses through selective targeting of a subclass of suppressor T cells [14]; suppressor T cells are now referred to as Treg cells. Harada and Makino performed the first experiment that demonstrated that CY promotes the onset of DM in NOD mice [15]. Charlton *et al.* subsequently confirmed that CY accelerates T1DM development in NOD mice and that this phenomenon could be prevented through an increase in the amount of mononuclear cells [16]. The principal mechanism elicited by CY in this “acceleration model” was related to the depletion of a Treg cell population [5–8]. Specifically, it seems that CY induces a selective apoptotic loss of CD4^+^CD25^+^FoxP3^+^ Treg cells from peripheral lymphoid tissues and the pancreas. Those Treg cells are involved in the suppression of the accumulation and function of effector T cells that attack pancreatic B cells [5, 7]. Furthermore, CY could directly increase the number of interferon (IFN)-α producer T helper type 1 (Th1)-like cells capable of inducing islet destruction [17] and indirectly accelerate insulitis through enhancing IFN-γ secretion [18]. These mechanisms support the appearance of autoimmune diabetes, requiring a life-long use of insulin.

In our patient, traditional islet autoantibodies were negative, and she discontinued the use of insulin after 5 months. However, tests for other antibodies, such as anti-insulin receptor antibodies or Zn8, could not be performed. We suspect that CY could have precipitated hyperglycemia by a cytotoxicity mechanism, but other non-identified mechanisms may also have played a role. We also hypothesize that the effect may have been cytotoxic but not cytocidal, or that the surviving beta cells are currently enough to maintain a long-term normoglycemia and C-peptide production. The association of other factors predisposing to hyperglycemia cannot be completely ruled out in most humans, including our patient. After an exhaustive review of the literature, we only found one clinical case reported by Sharma *et al.* [19] of a 51-year-old woman with ductal invasive breast carcinoma who was treated with an epirubicin and CY (EC) regimen after surgery. After completion of four cycles of chemotherapy, she began to have clinical manifestations of diabetes, with a random glucose determination of 338 mg/dl and HbA1c of 12.4%. Interestingly, she also had no family history of diabetes and she improved both clinically and biochemically with glimepiride and metformin. As in our case, her history was not compatible with autoimmune diabetes. These authors also considered that there could be a possible relationship with CY use and the onset of DM [19]. We consider that the frequency of atypical behavior of diabetes associated with CY has been underdiagnosed for many reasons, one being that the severity of the disease leading to the use of CY is usually the focus of attention of physicians and diabetes becomes a lesser worry. Another reason may be that most cases present with milder forms of diabetes that do not trigger the alarm for atypical diabetes. Finally, the presence of other risk factors and drugs make it difficult to assign a specific value to the influence of CY in humans. More cases need to be published in order to increase the evidence of this influence.

## Conclusion

We present the case of a patient with a difficult to classify diabetes who lacked the traditional clinical or biochemical markers for the common types of diabetes. She developed insulin deficiency, with high insulin requirements and decreased serum C-peptide. After ruling out the most common causes for diabetes, we considered that her hyperglycemic crisis might be at least partially related to the CY treatment. Until further evidence is published, glucose monitoring of patients being treated with this drug may be useful, especially if other risk factors for hyperglycemia are present.

## Abbreviations

CII: Continuous insulin infusion
CY: Cyclophosphamide
DKA: Diabetic ketoacidosis
DM: Diabetes mellitus
EC: Epirubicin and cyclophosphamide
ED: Emergency department
HbA1c: Glycated hemoglobin
IFN: Interferon
LADA: Latent autoimmune diabetes of the adult
MODY: Maturity-onset diabetes of the young
NOD: Non-obese diabetic
PEPCK: Phosphoenolpyruvate carboxykinase
SLE: Systemic lupus erythematosus
SLEDAI: Systemic Lupus Erythematosus Disease Activity Index
T1DM: Type 1 diabetes mellitus
TAG: Triacylglycerol
Th1: T helper type 1
Treg cells: Suppressor T cells

## References

[1] American Diabetes Association. 2. Classification and Diagnosis of Diabetes: Standards of Medical Care in Diabetes–2018. Diabetes Care. 2018;41:S13–27. 10.2337/dc18-S002 29222373

[2] Brode S, Raine T, Zaccone P, Cooke A. Cyclophosphamide-induced type-1 diabetes in the NOD mouse is associated with a reduction of CD4+CD25+Foxp3+ regulatory T cells. J Immunol. 2006;177:6603–12. 10.4049/jimmunol.177.10.6603 17082572

[3] Demirci H, Cosar R, Ciftci O, Sari IK. Atypical diabetic ketoacidosis: case report. Balkan Med J. 2015;32:124–6. 10.5152/balkanmedj.2015.15123 PMC4342127 25759786

[4] Kaur S, Tan WL, Soo C, Cheung CC, Stewart J, Reddy S. An immunohistochemical study on the distribution and frequency of T regulatory cells in pancreatic islets of NOD mice during various stages of spontaneous and cyclophosphamide-accelerated diabetes. Pancreas. 2010;39:1024–33. 10.1097/MPA.0b013e3181da9037 20442677

[5] Reddy S, Bradley J, Ginn S, Pathipati P, Ross JM. Immunohistochemical study of caspase-3-expressing cells within the pancreas of non-obese diabetic mice during cyclophosphamide-accelerated diabetes. Histochem Cell Biol. 2003;119:451–61. 10.1007/s00418-003-0537-0 12802593

[6] Caquard M, Ferret-Bernard S, Haurogne K, Ouary M, Allard M, Jegou D, Bach JM, Lieubeau B. Diabetes acceleration by cyclophosphamide in the non-obese diabetic mouse is associated with differentiation of immunosuppressive monocytes into immunostimulatory cells. Immunol Lett. 2010;129:85–93. 10.1016/j.imlet.2010.01.009 20144655

[7] Antkowiak PF, Stevens BK, Nunemaker CS, McDuffie M, Epstein FH. Manganese-enhanced magnetic resonance imaging detects declining pancreatic beta-cell mass in a cyclophosphamide-accelerated mouse model of type 1 diabetes. Diabetes. 2013;62:44–8. 10.2337/db12-0153 PMC3526033 22933107

[8] Gehi A, Webb A, Nolte M, Davis J Jr. Treatment of systemic lupus erythematosus-associated type B insulin resistance syndrome with cyclophosphamide and mycophenolate mofetil. Arthritis Rheum. 2003;48:1067–70. 10.1002/art.10879 12687550

[9] Yang H, Zhao J, Li Y, Lv F, Zhang S, Li Y. Successful treatment of type B insulin resistance with mixed connective tissue disease by pulse glucocorticoids and cyclophosphamide. J Diabetes Investig. 2017;8:626–8. 10.1111/jdi.12619 PMC5497044 28084020

[10] Ferreira GN, Rossi-Valentim R, Buzelle SL, Paula-Gomes S, Zanon NM, Garofalo MAR, Frasson D, Navegantes LCC, Chaves VE, Kettelhut IDC. Differential regulation of glyceroneogenesis by glucocorticoids in epididymal and retroperitoneal white adipose tissue from rats. Endocrine. 2017;57:287–97. 10.1007/s12020-017-1315-4 28555305

[11] Stratakis CA. Skin manifestations of Cushing’s syndrome. Rev Endocr Metab Disord. 2016;17:283–6. 10.1007/s11154-016-9399-3 PMC5181654 27943005

[12] Nyenwe EA, Kitabchi AE. The evolution of diabetic ketoacidosis: An update of its etiology, pathogenesis and management. Metabolism. 2016;65:507–21. 10.1016/j.metabol.2015.12.007 26975543

[13] Matos M, Park R, Mathis D, Benoist C. Progression to islet destruction in a cyclophosphamide-induced transgenic model: a microarray overview. Diabetes. 2004;53:2310–21. 10.2337/diabetes.53.9.2310 15331540

[14] Ahlmann M, Hempel G. The effect of cyclophosphamide on the immune system: implications for clinical cancer therapy. Cancer Chemother Pharmacol. 2016;78:661–71. 10.1007/s00280-016-3152-1 27646791

[15] Harada M, Makino S. Promotion of spontaneous diabetes in non-obese diabetes-prone mice by cyclophosphamide. Diabetologia 1984;27:604–6. 10.1007/BF00276978 6530055

[16] Charlton B, Bacelj A, Slattery RM, Mandel TE. Cyclophosphamide-induced diabetes in NOD/WEHI mice. Evidence for suppression in spontaneous autoimmune diabetes mellitus. Diabetes 1989; 38: 441–7. 10.2337/diab.38.4.441 2522407

[17] Campbell IL, Kay TW, Oxbrow L, Harrison LC. Essential role for interferon-gamma and interleukin-6 in autoimmune insulin-dependent diabetes in NOD/Wehi mice. J Clin Invest. 1991;87:739–42. 10.1172/JCI115055 PMC296368 1899431

[18] Ablamunits V, Quintana F, Reshef T, Elias D, Cohen IR. Acceleration of autoimmune diabetes by cyclophosphamide is associated with an enhanced IFN-gamma secretion pathway. J Autoimmun. 1999;13:383–92. 10.1006/jaut.1999.0331 10585754

[19] Sharma PK, Misra AK, Singh V, Gupta A, Saroha S, Singh S. Cyclophosphamide and epirubicin-induced diabetes mellitus in breast cancer: A rare occurrence. J Pharmacol Pharmacother. 2016;7:146–8. 10.4103/0976-500X.189684 PMC5020776 27651713

